# Pulled-up race outcomes as welfare-relevant surveillance indicators in United States Steeplechase horses, 2023–2025

**DOI:** 10.3389/fvets.2026.1910221

**Published:** 2026-10-02

**Authors:** Jeffrey S. Markowitz

**Affiliations:** Independent Researcher, Princeton Junction, NJ, United States

**Keywords:** epidemiology, non-completion, pulled up, racehorse welfare, race-result data, return to racing, steeplechase racing, time-to-event analysis

## Abstract

**Introduction:**

Pulled-up race outcomes are routinely recorded but are nonspecific, potentially reflecting fatigue, poor performance, footing, rider judgment, suspected injury, or physiological compromise. We evaluated their association with subsequent participation disruption in United States steeplechase horses.

**Methods:**

National Steeplechase Association Race Results Reports from 2023 to 2025 were analyzed retrospectively at the horse-start level. Outcomes were time to the next later-date start, ≥60-day interruption, ≥183-day absence, and no recorded later-date start. Cox models were stratified by race type with horse-clustered robust covariance; binary outcomes used horse-clustered generalized estimating equations. Sensitivity analyses excluded November starts because racing is seasonal from March through November.

**Results:**

Among 3,070 starts by 710 horses, 367 (11.95%) were pulled up, involving 277 horses (39.0%). No later-date start was recorded after 37.6% of pulled-up and 21.2% of non-pulled-up starts. Among returners, median time to the next start was 42 versus 35 days; Kaplan–Meier medians for all starts were 148 versus 45 days. Pulled-up status was associated with a lower recorded return rate (hazard ratio 0.589, 95% confidence interval 0.515–0.675). Race-type-adjusted odds ratios were 2.10 (1.67–2.64) for ≥60-day interruption and 2.36 (1.80–3.10) for ≥183-day absence. Associations persisted after excluding November starts.

**Discussion:**

Pulled-up outcomes were associated with participation disruption, although clinical reasons were unavailable. They may help prioritize follow-up and longitudinal surveillance but cannot replace veterinary examination or clinical records.

## Introduction

Pulled-up race outcomes are a routine part of official race reporting but have received limited focused attention as epidemiological exposures. In official results, a pulled-up designation generally indicates that the rider intentionally stopped or withdrew the horse before the finish. It is distinct from a fall or lost-rider event and can reflect poor performance, fatigue, respiratory or metabolic compromise, suspected injury, adverse footing or weather, precautionary rider judgment, or other race-day circumstances. These heterogeneous causes mean that pulled-up status should be evaluated as an operational surveillance marker, not as a diagnosis or a uniform welfare event. Prior research has generally included pulled-up events within broader failure-to-finish categories (1).

Steeplechase racing is a useful setting in which to study pulled-up outcomes. Compared with flat racing, it involves longer distances, obstacles, variable terrain, and a limited seasonal calendar. Studies from other jurisdictions have documented falls and fatalities in jump racing (2–4), but less is known about non-completion outcomes that do not involve a fall or lost rider. Tanner et al. studied failure to finish in New Zealand Thoroughbred flat racing, where pulled-up, fell, and lost-rider outcomes contributed to a broader endpoint (1). Legg et al. evaluated risk factors for horse falls in New Zealand jumps racing and considered pulling up within a wider race-safety context (3). Neither study followed later participation after a pulled-up event as a distinct exposure.

Racehorse welfare surveillance includes indicators with different strengths. Fatality and fall surveillance identifies severe events but does not capture all non-catastrophic concerns. Veterinary examination, diagnosis, treatment, and recovery records are more specific, but they are not routinely available in public datasets. Participation measures such as delayed return and prolonged absence are accessible and longitudinal, but they cannot establish why a horse stopped racing or whether its welfare was impaired. Their value lies in identifying patterns that may warrant investigation, not in assigning a clinical state to an individual horse.

Regulatory context also affects interpretation. Current National Steeplechase Association rules require the race-day veterinarian to examine a horse when its physical condition is questioned and any horse that appears physically distressed during or at the finish. Horses placed on the Stewards’ or Veterinarian’s List, or otherwise not in sound racing condition, are ineligible to race (5). The reviewed rules do not state that a PU result alone automatically triggers examination, placement on a list, or a specified stand-down period. Additional commission or venue requirements may apply at recognized meetings. Race-result data therefore cannot be assumed to contain or represent any veterinary review that may occur after a race.

Public race records can complement direct surveillance by documenting longitudinal participation. They rarely contain diagnoses, treatment information, or recovery trajectories, but they can show whether and when a horse returned to racing (6). Linked administrative and incident records can add clinical context. Morrice-West et al., for example, linked horse incidents and fatalities with jockey falls and injuries in Australian flat racing (7). That design is more clinically informative than race-result surveillance alone and provides a model for future validation of pulled-up outcomes. Time-loss and return-to-participation measures are also used in sports epidemiology when direct clinical information is incomplete (8, 9), although their meaning depends on the population and competition calendar.

This study examined whether pulled-up race outcomes were associated with subsequent participation disruption in United States steeplechase horses during 2023–2025. The outcomes were time to the next start, a ≥ 60-day interruption, a ≥ 183-day absence, and no recorded later-date start. The study tested whether a routinely recorded pulled-up designation identifies index starts followed by greater participation disruption. It did not infer injury, disease, pain, or clinical welfare status from the race result.

## Materials and methods

### Study design and data source

This retrospective observational study used publicly available Race Results Reports from National Steeplechase Association races conducted from March 24, 2023, through November 23, 2025. The analytic window comprised three complete contemporary seasons for which race-result reports had been assembled and audited for this project. Earlier NSA race-result reports were available but were outside the defined study window and were not included in the project dataset. After audit and cleaning, the analytic dataset contained 3,070 eligible horse-starts.

Each start was treated as an index observation because the official pulled-up designation is assigned to a specific race start, and each start begins a new interval to the next later-date start. Horses could contribute more than one index start. The estimands are therefore start-level associations rather than horse-level lifetime risks; inferential models accounted for within-horse dependence.

### Data extraction and quality control

The analytic fields were race date, course or meet, race number, horse, rider, race type, distance, track condition, field size, and official outcome indicators. Dates stored as Excel serial values were converted to SAS dates. The file was checked for missing horse identifiers, dates, calendar year, race type, and pulled-up status; none of these core fields was missing. Additional completeness checks assessed distance, field size, track condition, course or meet, horse sex, and foaling date. SAS quality-control procedures were also used throughout data preparation and analysis to verify record counts and frequency distributions, identify duplicate or inconsistent records, assess ranges and missing values, and confirm that derived variables and analytic classifications produced the expected totals. Key counts and distributions were rechecked after data transformations and during revision of the analyses. The results of these checks were used to determine whether the available variables could support descriptive or adjusted analyses.

Distance, field size, track condition, and course or meet were complete and were retained for cohort description and quality control. Horse sex and foaling date were missing for 2,889 starts (94.1%) and could not support reliable age- or sex-adjusted analyses. Veterinary diagnosis, injury history, treatment, training status, and complete prior racing history were unavailable. Trainer, course, distance, field size, and track condition were not included in the association models because a comprehensive etiologic risk model was not prespecified. Their omission, and the absence of clinical horse-level variables, leave residual confounding.

Calendar year was examined descriptively to characterize variation in pulled-up frequency across the three seasons and was not used as a stratification variable in the association models. Race type was also summarized descriptively and was incorporated separately in the association analyses as described below. Month was used to define the prespecified November sensitivity analysis because of the seasonal racing calendar. Course, track condition, distance, and field size were not screened for statistical significance, and the study does not imply adjustment for those factors.

### Exposure definition

The primary exposure was the official outcome code PU, indicating pulled up. Starts coded PU were classified as pulled up, and all other eligible starts were the comparison group. The official codes F, indicating that the horse fell, and LR, indicating a lost rider, were separately coded and were not recoded as pulled-up starts. No alternative text labels were mapped to PU, and no clinical cause was inferred from the code.

Race type was coded as fence or hurdle versus timber. These groups differ in obstacle type and race characteristics; timber races are generally conducted over larger fixed obstacles and longer distances.

### Participation outcomes

The primary time-to-event outcome was the number of days from the index start to the horse’s next recorded start on a later calendar date. A ≥ 60-day interruption represented an operational interval of approximately 2 months. A ≥ 183-day absence represented an interval of approximately 6 months. These prespecified thresholds were intended to distinguish increasing durations of participation disruption; they are not clinically validated injury or recovery cut points. No recorded later-date start during available follow-up was summarized descriptively.

The outcomes were interpreted as indirect, welfare-relevant participation measures. They were not treated as measures of diagnosis, pain, injury severity, affective state, treatment, or clinical recovery.

### Censoring and outcome eligibility

For Kaplan–Meier and Cox analyses, return to racing was a subsequent recorded start on a later calendar date. Starts without a later-date start were right-censored on November 23, 2025, the final race date in the dataset. Potential follow-up was the number of calendar days from the index start to that administrative end date. Censoring indicates that return was not observed before follow-up ended; it does not establish retirement, death, transfer to another discipline, continued training, clinical recovery, or persistent illness. Same-day starts, if present, were not counted as later returns.

For the ≥60-day outcome, a start was classifiable if the horse returned before day 60 or at least 60 days of potential follow-up remained. Return before day 60 was classified as no interruption; return on or after day 60, or no recorded return with at least 60 days of potential follow-up, was classified as an interruption. Starts with no recorded return and fewer than 60 potential follow-up days were excluded.

For the ≥183-day outcome, index starts were restricted to 2023–2024 to ensure adequate observation. Return before day 183 was classified as no extended absence; return on or after day 183, or no recorded return, was classified as an extended absence. Time-to-event analyses included all 3,070 starts. The ≥60-day analysis included 2,831 classifiable starts, and the ≥183-day analysis included 1,962 starts.

National Steeplechase Association racing is conducted from March through November. Late-season starts have fewer opportunities for another start before administrative censoring. All principal models were therefore repeated after November starts were excluded. The November-excluded time-to-event analysis included 2,614 starts.

### Descriptive follow-up review

To describe observable outcomes among horses with apparent prolonged absence after a pulled-up event, publicly available Equibase records (10) were reviewed for horses whose final recorded National Steeplechase Association start during 2023–2024 was coded PU and who had no recorded National Steeplechase Association start during 2025. The review classified later jump-racing return, later flat-racing return, no recorded return through June 1, 2026, or another publicly documented outcome. The review was descriptive and was not used in the association models.

### Statistical analysis

Descriptive statistics summarized exposure distribution, time to the next start, and binary participation outcomes. Means, medians, interquartile ranges, and observed ranges were reported as appropriate. Pearson chi-square tests described differences in pulled-up frequency across calendar years and race types. Because individual horses could contribute multiple starts, these exposure-distribution tests were considered descriptive.

Kaplan–Meier methods estimated return-to-racing distributions for all starts, with starts lacking an observed return treated as administratively censored. Kaplan–Meier estimates were interpreted descriptively; unclustered log-rank and returner-only rank-sum *p* values were not used for inference. Cox proportional hazards models estimated hazard ratios and 95% confidence intervals for time to the next start. Return was the event, so a hazard ratio below 1.0 indicated a lower rate of recorded return. Robust sandwich covariance estimates were clustered by horse.

The proportional-hazards assumption was evaluated with supremum tests. Race type violated proportional hazards when entered as a conventional covariate (*p* < 0.0001), whereas pulled-up status did not violate the assumption in the race-type-stratified model (*p* = 0.146). The principal Cox model was therefore stratified by race type and included pulled-up status as the exposure, with covariance clustered by horse.

Binary outcomes were modeled with generalized estimating equations using a binomial distribution, logit link, independent working correlation, horse as the clustering variable, and empirical standard errors. Unadjusted and race-type-adjusted odds ratios were estimated for the ≥60-day and ≥183-day outcomes. The November-excluded analyses used the same race-type-stratified Cox and race-type-adjusted generalized estimating-equation specifications. Analyses were conducted in SAS version 9.4.

## Results

### Study sample and pulled-up exposure distribution

The study included 3,070 starts by 710 horses. Overall, 367 starts (11.95%; 119.5 per 1,000 starts) were designated as pulled up. Of 710 unique horses, 277 (39.0%) had at least one pulled-up start. Among these 277 horses, 198 had one pulled-up start, 69 had two, nine had three, and one had four during the study period.

There were 119 pulled-up starts in 2023, 137 in 2024, and 111 in 2025. Pulled-up frequency differed modestly by year (Pearson chi-square = 6.90, df = 2, *p* = 0.032) but not by race type (chi-square = 0.27, df = 1, *p* = 0.60). Table 1 summarizes the cohort and exposure distribution.

**Table 1:** Study sample and pulled-up exposure distribution among United States steeplechase horse starts, 2023–2025 (*n* = 3,070).

Measure	Value
Total starts	3,070
Unique horses	710
Horses with ≥1 pulled-up start	277/710 (39.0%)
Pulled-up starts	367 (11.95%)
Pulled-up rate	119.5 per 1,000 starts
Non-pulled-up starts	2,703 (88.05%)
Horses with 1/2 / 3/4 pulled-up starts	198/69 / 9/1
Fence or hurdle starts	2,531 (82.44%)
Timber starts	539 (17.56%)
Pulled-up starts in 2023	119/959 (12.41%)
Pulled-up starts in 2024	137/1,003 (13.66%)
Pulled-up starts in 2025	111/1,108 (10.02%)
Pulled-up fence or hurdle starts	299/2,531 (11.81%)
Pulled-up timber starts	68/539 (12.62%)

Pulled-up frequency differed by year (Pearson chi-square = 6.90, df = 2, *p* = 0.032) but not by race type (chi-square = 0.27, df = 1, *p* = 0.60). Year and race-type tests describe the exposure distribution and do not account for repeated starts by the same horse.

### Time to the next start

A later-date start was observed after 229 of 367 pulled-up starts (62.4%) and 2,131 of 2,703 non-pulled-up starts (78.8%). Conversely, no later-date start was recorded during available follow-up after 138 pulled-up starts (37.6%) and 572 non-pulled-up starts (21.2%). These proportions are descriptive and do not classify the reason for absence.

Among index starts followed by an observed return, median time to the next start was 42 days after a pulled-up start and 35 days after a non-pulled-up start. Corresponding means were 95.9 and 80.5 days, and interquartile ranges were 20–148 and 20–117 days. These summaries are conditional on an observed return.

When all starts were included and unobserved returns were treated as censored, Kaplan–Meier median time to the next start was 148 days after pulled-up starts and 45 days after non-pulled-up starts. Fewer than 75% of pulled-up starts were followed by an observed return, so the 75th percentile could not be estimated; its lower 95% confidence bound was 713 days. The non-pulled-up 75th percentile was 167 days. These values describe the observed return process and are not estimates of clinical recovery time.

In the principal race-type-stratified Cox model with horse-clustered robust covariance, pulled-up status was associated with a lower rate of recorded return (hazard ratio 0.589, 95% confidence interval 0.515–0.675; *p* < 0.0001). The proportional-hazards test for pulled-up status was not significant (*p* = 0.146). Table 2 presents the time-to-event results.

**Table 2:** Time to the next start after pulled-up and non-pulled-up United States steeplechase horse starts, 2023–2025.

Outcome	Non-pulled-up	Pulled-up	Comparison
Observed later-date start	2,131/2,703 (78.84%)	229/367 (62.40%)	Descriptive
No recorded later-date start	572/2,703 (21.16%)	138/367 (37.60%)	Descriptive
Mean days to next start among returners	80.5	95.9	Descriptive
Median days to next start among returners	35	42	Descriptive
Interquartile range among returners	20–117	20–148	Descriptive
Kaplan–Meier median, all starts	45 days	148 days	Descriptive
Kaplan–Meier 75th percentile	167 days	Not reached; lower 95% CI 713 days	Descriptive
Race-type-stratified Cox model with horse-clustered covariance	Reference	HR 0.589 (95% CI 0.515–0.675)	*p* < 0.0001
Proportional-hazards test for pulled-up status	–	*p* = 0.146	Assumption not rejected

CI, confidence interval; HR, hazard ratio. Returner-only summaries are conditional on an observed later-date start. Kaplan–Meier estimates include all starts and treat starts without an observed return as administratively censored. The Cox model is stratified by race type and uses robust covariance clustered by horse. Unclustered rank-sum and log-rank *p* values are not reported.

### Prolonged interruption and extended absence

Among starts eligible for the ≥60-day endpoint, 212 of 336 pulled-up starts (63.1%) and 1,119 of 2,495 non-pulled-up starts (44.9%) were followed by an interruption. The horse-clustered unadjusted generalized estimating-equation odds ratio was 2.10 (95% confidence interval 1.67–2.64), and the race-type-adjusted odds ratio was 2.10 (1.67–2.64); both *p* < 0.0001. In the adjusted model, timber starts also had higher odds of a ≥ 60-day interruption than fence or hurdle starts (odds ratio 1.36, 95% confidence interval 1.17–1.57; *p* < 0.0001).

Among starts eligible for the ≥183-day endpoint, 108 of 256 pulled-up starts (42.2%) and 405 of 1,706 non-pulled-up starts (23.7%) were followed by an extended absence. The horse-clustered unadjusted odds ratio was 2.34 (95% confidence interval 1.79–3.07), and the race-type-adjusted odds ratio was 2.36 (1.80–3.10); both *p* < 0.0001. Timber starts also had higher adjusted odds of a ≥ 183-day absence (odds ratio 1.84, 95% confidence interval 1.45–2.33; *p* < 0.0001). Table 3 summarizes the binary outcomes.

**Table 3:** Binary participation outcomes after pulled-up and non-pulled-up United States steeplechase horse starts, 2023–2025.

Outcome	Non-pulled-up	Pulled-up	Unadjusted GEE OR (95% CI)	Race-type-adjusted GEE OR (95% CI)
≥60-day interruption	1,119/2,495 (44.9%)	212/336 (63.1%)	2.10 (1.67–2.64)	2.10 (1.67–2.64)
≥183-day absence	405/1,706 (23.7%)	108/256 (42.2%)	2.34 (1.79–3.07)	2.36 (1.80–3.10)
No recorded later-date start	572/2,703 (21.2%)	138/367 (37.6%)	Not modeled	Not modeled

CI, confidence interval; GEE, generalized estimating equation; OR, odds ratio. GEE models use horse as the clustering variable and empirical standard errors. The ≥60-day analysis included 2,831 classifiable starts. The ≥183-day analysis included 1,962 starts from 2023 to 2024. No recorded later-date start was evaluated descriptively.

### Late-season sensitivity analyses

November starts accounted for 15.0% of pulled-up starts and 14.8% of non-pulled-up starts. After November starts were excluded, pulled-up status remained associated with a lower rate of recorded return in the race-type-stratified, horse-clustered Cox model (hazard ratio 0.616, 95% confidence interval 0.534–0.712; *p* < 0.0001).

In the November-excluded, race-type-adjusted generalized estimating-equation models, pulled-up status remained associated with a ≥ 60-day interruption (odds ratio 2.07, 95% confidence interval 1.63–2.63) and a ≥ 183-day absence (odds ratio 2.08, 1.54–2.81); both *p* < 0.0001. Table 4 summarizes these sensitivity analyses.

**Table 4:** Sensitivity analyses excluding November starts among United States steeplechase horse starts, 2023–2025.

Outcome	Non-pulled-up	Pulled-up	Comparison or effect estimate
Race-type-stratified Cox model with horse-clustered covariance	Reference	–	HR 0.616 (95% CI 0.534–0.712)
≥60-day interruption	935/2,241 (41.7%)	180/301 (59.8%)	Adjusted GEE OR 2.07 (95% CI 1.63–2.63)
≥183-day absence	346/1,481 (23.4%)	86/223 (38.6%)	Adjusted GEE OR 2.08 (95% CI 1.54–2.81)

CI, confidence interval; GEE, generalized estimating equation; HR, hazard ratio; OR, odds ratio. The Cox model is stratified by race type with horse-clustered robust covariance; GEE estimates are race-type adjusted and horse clustered. All modeled pulled-up associations had *p* < 0.0001.

### Descriptive follow-up of the selected pulled-up subgroup

The descriptive Equibase review included 83 horses whose final recorded National Steeplechase Association start during 2023–2024 was coded PU and who had no recorded National Steeplechase Association start during 2025. Five horses (6.0%) later returned to jump racing and four (4.8%) returned to flat racing during 2026. 74 (89.2%) had no recorded return through June 1, 2026. Of these 74 horses, one died following the index race.

## Discussion

### Principal findings

Pulled-up race outcomes were consistently associated with subsequent participation disruption in this three-season United States steeplechase cohort. Pulled-up starts were followed by a lower rate of recorded return and approximately twofold higher odds of both a ≥ 60-day interruption and a ≥ 183-day absence. The estimates changed little after within-horse dependence was addressed and after November starts were excluded.

These associations do not show that pulling up caused later absence or that each pulled-up horse was injured. Pulled-up status can reflect horse condition, footing, weather, rider judgment, poor performance, precautionary management, or other factors that also influence later participation. The race-result data contained no diagnosis, examination result, pain measure, treatment, or recovery trajectory. The findings therefore describe an association between an operational race code and later participation, not a clinical outcome.

The distinction between returners-only summaries and the all-start time-to-event analysis is important. Among observed returners, median time to the next start differed by 7 days. The Kaplan–Meier and Cox analyses showed a larger difference because they retained follow-up from starts without an observed later return. Censoring does not mean that a horse never returned; it means only that no later start was observed before the administrative end date.

### Comparison with prior literature

Earlier New Zealand studies examined related but different endpoints. Tanner et al. analyzed failure to finish in Thoroughbred flat racing and combined several non-completion outcomes within the broader endpoint (1). Legg et al. evaluated risk factors for horse falls in jumps racing, with pulling up considered in the race-safety setting rather than as a distinct predictor of later participation (3). Gibson et al. described race-level incidents and fatalities in New Zealand racing (4, 6). The present study extends this literature by treating pulled-up status as a separate index exposure and following subsequent starts.

The Australian linked-record study by Morrice-West et al. should be viewed as a future direction rather than a direct methodological comparison (7). That study linked racehorse incidents and fatalities with jockey falls and injuries. Similar linkage of pulled-up results to race-day examinations, incident reports, veterinarian-list placement, diagnoses, treatment, and return clearance would be required to determine the clinical meaning of the surveillance signal observed here.

### Animal welfare and surveillance implications

Pulled-up status may be useful because it is routinely recorded, easy to identify, and associated with later participation disruption. Its practical role would be to prioritize assessment, not to classify injury or welfare impairment. A useful surveillance process could begin with a standardized reason-for-pulling-up category, followed by race-day veterinary assessment when indicated, documentation of relevant clinical or contextual findings, short-term follow-up, and review of readiness to return. Aggregate monitoring could then examine whether follow-up occurred and whether prolonged absence, retirement, transfer, or death was documented.

Relevant welfare components include lameness or injury, respiratory or metabolic compromise, fatigue, pain or distress, footing and weather, treatment, recovery, and readiness to race again. Public participation data cannot measure these components directly. A pulled-up flag should therefore complement veterinary examination, trainer observation, and regulatory review. It should not replace them or be used as a punitive marker in the absence of clinical context.

Current NSA rules already provide for veterinary examination when a horse’s condition is questioned or it appears in distress, and they restrict horses placed on official lists (5). The present findings do not establish whether these procedures were applied after individual pulled-up starts or whether they improved outcomes. The results instead support evaluating whether standardized documentation and record linkage could make existing follow-up more consistent and assessable. Ontario’s Thoroughbred rules provide a jurisdiction-specific example: horses considered unfit by a Commission or Official Veterinarian are placed on the Veterinarians’ List and must satisfy applicable clearance requirements before returning to competition (11). This comparison illustrates a formal follow-up mechanism but should not be generalized across countries or racing systems.

### Limitations

The principal limitation is the absence of clinical and veterinary information. The study could not determine why a horse was pulled up or why a later start was delayed or absent. Injury, illness, fatigue, precautionary rest, training changes, race availability, owner or trainer decisions, retirement, transfer to another discipline, and death are all possible. Participation disruption is therefore an indirect welfare-relevant indicator, not a measure of pain, injury severity, affective state, or clinical welfare status.

The observational design precludes causal inference. Important horse-level variables, including reliable age, sex, prior history, and veterinary status, were unavailable. Trainer, course, distance, track condition, and field size were not included in the association models. The estimates may therefore reflect residual confounding by horse condition, race circumstances, management, or scheduling.

Some horses contributed multiple starts. Horse-clustered robust Cox covariance and generalized estimating equations addressed within-horse dependence for the modeled outcomes, but the results remain start-level associations and should not be interpreted as lifetime horse-level probabilities. Kaplan–Meier estimates were retained as descriptive summaries because conventional curves do not account for repeated starts from the same horse.

Administrative censoring and the seasonal calendar can misclassify the meaning of no recorded later-date start. A censored observation may represent retirement, death, discipline transfer, continued training, lack of an available race, or an outcome outside the dataset. Restricting the ≥183-day analysis to 2023–2024 and excluding November starts in sensitivity analyses reduced, but did not eliminate, this uncertainty.

The Equibase follow-up review was restricted to horses whose final recorded National Steeplechase Association start during 2023–2024 was coded PU and who had no National Steeplechase Association start during 2025. It had no comparable non-pulled-up group and therefore cannot estimate excess non-return or mortality risk. Public records may also omit retirement, continued training, private veterinary outcomes, sales, exports, or participation outside the records reviewed.

Finally, the cohort covered three seasons in a small, seasonal United States steeplechase system with a specific regulatory context. Effect magnitudes may not apply to flat racing, larger year-round programs, or jump-racing jurisdictions with different result codes, veterinary reporting, calendars, and return opportunities.

## Conclusion

Pulled-up race outcomes were associated with a lower rate of recorded return and higher odds of prolonged participation disruption in United States steeplechase horses. These start-level associations persisted after within-horse dependence was addressed and after November starts were excluded. They do not establish the cause of pulling up or confirm injury, disease, pain, or impaired welfare in an individual horse.

Systematic monitoring of pulled-up outcomes and subsequent participation may help prioritize horses for follow-up assessment and broaden surveillance beyond falls and fatalities. Direct veterinary evaluation remains necessary to determine clinical significance. Future studies should add standardized reason-for-pulling-up categories, link race results with examinations and veterinarian-list records, include suitable non-pulled-up comparison groups, replicate the analysis in other seasons and jurisdictions, and test whether targeted follow-up changes clinical or participation outcomes.

## Data Availability

The original contributions presented in the study are included in the article/Supplementary material, further inquiries can be directed to the corresponding author.
